# Investigation of energy metabolic dynamism in hyperthermia-resistant ovarian and uterine cancer cells under heat stress

**DOI:** 10.1038/s41598-021-94031-9

**Published:** 2021-07-19

**Authors:** Taisei Kanamori, Natumi Miyazaki, Shigeki Aoki, Kousei Ito, Akihiro Hisaka, Hiroto Hatakeyama

**Affiliations:** 1 Laboratory of Clinical Pharmacology and Pharmacometrics, Graduate School of Pharmaceutical Sciences, Chiba University, 1-8-1, Inohana, Chuou-ku, Chiba, 260-0856 Japan; 2 Laboratory of Biopharmaceutics, Graduate School of Pharmaceutical Sciences, Chiba University, 1-8-1, Inohana, Chuou-ku, Chiba, 260-0856 Japan

**Keywords:** Cancer, Cell biology

## Abstract

Despite progress in the use of hyperthermia in clinical practice, the thermosensitivity of cancer cells is poorly understood. In a previous study, we found that sensitivity to hyperthermia varied between ovarian and uterine cancer cell lines. Upon hyperthermia, glycolytic enzymes decreased in hyperthermia-resistant SKOV3 cells. However, the mechanisms of glycolysis inhibition and their relationship with thermoresistance remain to be explored. In this study, metabolomic analysis indicated the downregulation of glycolytic metabolites in SKOV3 cells after hyperthermia. Proteomic and pathway analyses predicted that the ubiquitin pathway was explicitly activated in resistant SKOV3 cells, compared with hyperthermia-sensitive A2780 cells, and STUB1, a ubiquitin ligase, potentially targeted PKM, a glycolytic rate-limiting enzyme. PKM is degraded via ubiquitination upon hyperthermia. Although glycolysis is inactivated by hyperthermia, ATP production is maintained. We observed that oxygen consumption and mitochondrial membrane potential were activated in SKOV3 cells but suppressed in A2780 cells. The activation of mitochondria could compensate for the loss of ATP production due to the suppression of glycolysis by hyperthermia. Although the physiological significance has not yet been elucidated, our results demonstrated that metabolomic adaptation from the Warburg effect to mitochondrial oxidative phosphorylation could contribute to thermoresistance in ovarian and uterine cancer cells.

## Introduction

Therapeutic hyperthermia involves raising the temperature of the tumor tissue to 40–43 °C. These high temperatures can damage and kill cancer cells with minimal injury to normal tissues^1^. Hyperthermia increases the expression of heat-shock proteins (HSPs) and accelerates the release of intracellular damage-associated molecular patterns, including HSPs, adenosine triphosphate (ATP), and high mobility group box 1, which activate the immune system against cancer cells. This cell death pathway is termed immunogenic cell death^2^. In addition to the direct killing effect, hyperthermia sensitizes cancer cells to anticancer drugs^3^. Therefore, hyperthermia has attracted increased interest, and clinical trials have been conducted^4–6^. Hyperthermic intraperitoneal chemotherapy (HIPEC) with chemotherapy has been reported to prolong the overall survival of patients with ovarian cancers^6^. However, no survival benefit was observed in patients with recurrent ovarian cancer after hyperthermic treatment^7^. This suggests that hyperthermic treatment might be insufficient for the advanced stages of ovarian cancer. Thus, the efficacy of hyperthermic treatment should be improved by investigating the response of cancer cells to hyperthermia.

Recently, we reported that some ovarian and uterine cancer cells show resistance to hyperthermia, suggesting that these cancer cells cannot be killed by hyperthermic treatment^8^. Therefore, it is possible that targeting the mechanisms behind thermoresistance can lead to more efficient hyperthermic treatment in the clinic. In a previous report, connective tissue growth factor (CTGF) inhibition sensitized resistant cells to hyperthermia. Proteomic analysis revealed that glycolysis-related enzymes were downregulated after hyperthermia in resistant human ovarian SKOV3 cells. However, the detailed mechanisms by which glycolytic enzymes are downregulated and the effect of this downregulation on cellular metabolism has not yet been elucidated. In the present study, we evaluated the cellular metabolic alterations in SKOV3 cells using capillary electrophoresis time-of-flight mass spectrometry (CE-TOFMS)^9–11^. We also reanalyzed the proteomics data to explore the mechanisms underlying the downregulation of glycolytic enzymes and explored the metabolic adaptation after the downregulation of the glycolytic pathway.

## Methods

### Materials

The following primary antibodies were used for western blotting: PKM1/2 (1:3000, ab137791, Abcam, Cambridge, UK), anti-STUB1 (1:10,000, ab134064, Abcam, Cambridge, UK), anti-Ub (1:3000, # 646302, Biolegend, San Diego, CA, USA), and ββ-actin (1:10,000; Sigma-Aldrich, St. Louis, MO, USA). Plasmid DNA encoding ATeam (ATeam1.03-nD/nA/pcDNA3, #51958) was purchased from Addgene (Watertown, MA, USA). 2-Deoxy-D-glucose (2-DG) was purchased from Sigma-Aldrich (St. Louis, MO, USA).

### Cell culture

Human ovarian cancer A2780 cells were purchased from the European Collection of Authenticated Cell Culture (Wiltshire, UK). Human ovarian cancer SKOV3 and uterine KLE cells were purchased from the American Type Culture Collection (Manassas, VA, USA). Human uterine cancer Hec-1A cells were purchased from the Japanese Collection of Research Bioresources Cell Bank (Osaka, Japan). Cells were cultured in RPMI 1640 (Sigma-Aldrich) supplemented with 10% fetal bovine serum (Biowest, Paris, France) and 1% penicillin and streptomycin (Nacalai Tesque, Tokyo, Japan) in 5% CO_2_ at 37 °C.

### Heat shock of cancer cells

Cells were seeded in 6-cm dishes (2–4 × 10^5^ cells/3 mL of culture medium) or 10-cm dishes (1–1.5 × 10^6^ cells/10 mL of culture medium). The cells were heated in a cell culture chamber (C-140A; Blast, Kawasaki, Japan) for 1 h at the indicated temperatures, measured with a thermal imaging camera (E6; FLIR, Wilsonville, OR, USA), followed by incubation at 37 °C in a regular cell culture incubator 2.

### Protein expression analysis

Proteomics data that were quantitated by LC–MS/MS were obtained from a previous report^8^. Briefly, A2780 and SKOV3 were incubated either at 37 °C or at 46 °C for 1 h, followed by incubation at 37 °C for 4 h. Global expression changes in proteins were examined by TMT labeling and offline 2D-LC–MS/MS. Differentially expressed proteins were defined as having a Log2 (expression change) of > 0.5, between 37 °C and 46 °C cells, with a statistically significant Benjamin-Hochberg-adjusted P-value (A2780 < 0.2, SKOV3 < 0.05). Proteins that were specifically upregulated or downregulated in SKOV3 cells were extracted and compared with those in A2780 cells. The top canonical pathways associated with specifically upregulated and downregulated proteins in SKOV3 cells were identified using DAVID. The association of upregulated proteins with downregulated proteins in SKOV3 cells was analyzed using Ingenuity Pathway Analysis (IPA).

### Analysis of intracellular metabolites

Intracellular metabolites were extracted by chloroform–methanol extraction as mentioned previously^11^. Briefly, heated and non-heated cells were washed twice with 5% mannitol solution and then treated with methanol containing internal standards (H3304-1001; Human Metabolome Technologies). After adding deionized distilled water (DDW) and chloroform, the extract was centrifuged at 2300×*g* at 4 °C for 5 min and was then filtered through polyethersulfone (0.45 μm) (OD003C34; Nippon Genetics, Tokyo, Japan). The filtrate was concentrated by decompression centrifugation and dissolved in 25 μL of DDW before measurement. The concentrations of metabolites in the samples were measured using CE-TOFMS (Agilent Technologies, Santa Clara, CA, USA). The CE-TOFMS results were processed using MassHunter software (Quantitative analysis, Agilent Technologies). Fold changes in metabolites in the heated cells were calculated relative to those in the non-heated cells. Metabolites with a fold change greater than two were used for pathway analysis by MetaboAnalyst (https://www.metaboanalyst.ca/).

### Western blotting

Protein lysates were prepared from cultured ovarian cancer cells using a modified radioimmunoprecipitation assay buffer (Nacalai Tesque), 10 mM β-glycerophosphate, 1 mM ethylenediaminetetraacetic acid, and 1 mM sodium orthovanadate. Protein concentration in the lysates was determined using a BCA protein assay reagent kit. Protein lysates were separated by 10% sodium dodecyl sulfate–polyacrylamide gel electrophoresis (SDS-PAGE), transferred onto a nitrocellulose membrane, and blocked with 5% bovine serum albumin (BSA; Nacalai Tesque) in Tris-buffered saline with Tween 20 (TBST) for 1 h at room temperature. Membranes were probed with primary antibodies in 5% bovine serum albumin (BSA) in TBST overnight at 4 °C. The bands were then incubated with horseradish peroxidase-conjugated anti-mouse or anti-rabbit secondary antibodies (1:3000, Cell Signaling Technology, mouse: #7076, rabbit: #7074) for 1 h at room temperature. Blots were developed using an enhanced chemiluminescence detection kit (Can Get Signal; TOYOBO, Osaka, Japan).

### Immunoprecipitation

Protein lysates containing 300 µg protein were mixed with 2 µg of primary antibody (anti-PKM1/2, ab137791, Abcam, Cambridge, UK) and incubated for 1 h at 4 °C. Twenty microliters of suspended protein A-agarose (#sc2001, SANTA CRUZ, Santa Cruz, CA, USA) was added to the lysates and incubated overnight at 4 °C on a rotating device. The immunoprecipitates were collected by centrifugation at 1000 × g at 4 °C for 5 min. Pellets were washed with phosphate-buffered saline (PBS). After washing, the pellets were resuspended in 40 µL of electrophoresis sample buffer. The samples were analyzed using western blotting.

### Measurement of cellular oxygen consumption rate

The O_2_ consumption rate in cells was measured using a fluorescent oxygen probe (PreSens Sensor Dish Reader, Regensburg, Germany), as described previously^12^. O_2_ tension was monitored continuously every minute, and the concentration at time 0 was defined as 100%. The O_2_ consumption rate at time 0 was calculated using the differential calculus of the O_2_ change curve.

### Flow cytometric evaluation of mitochondrial membrane potential

Heated and non-heated cells were incubated for 30 min with 1 µM rhodamine 123 (CAYMAN chemical, Ann Arbor, MI, USA) dissolved in RPMI-1640. After incubation, the cells were washed and detached using trypsin-ethylenediaminetetraacetic acid. The cell pellets were washed twice with 1 mL PBS (Nissui Pharmaceutical, Tokyo, Japan). After centrifugation at 200 × g at 4 °C for 3 min and removing the supernatant, pellets were suspended in 200 µL of PBS. The suspension was passed through a 48-µm nylon mesh (Tokyo Garasu Kikai, Tokyo, Japan) to obtain a single-cell suspension. Cells were stained with 7-AAD (BioLegend) to exclude dead cells. Rhodamine 123 was detected at excitation, and emission wavelengths of 507 and 529 nm, respectively, and 7-AAD was detected at excitation and emission wavelengths of 540 nm and 650 nm, respectively, using a Novocyte flow cytometer (ACEA Biosciences, San Diego, CA, USA).

### ATeam transfection into cells

Plasmid DNA encoding ATeam1.03-nD was prepared using the ZymoPURE™ Plasmid Midiprep Kit (Zymo Research, Irvine, CA, USA). Cells were seeded in 10-cm dishes (2–3 × 10^6^ cells/10 mL of culture medium). Plasmid DNA was transfected using Lipofectamine 2000 (LFN2000; Thermo Fisher Scientific, Waltham, MA, USA) according to the manufacturer’s instructions. The cells were then washed with PBS, after which 9 mL of serum-free culture medium and 1 mL of LFN2000/plasmid DNA mixture containing 7 µg AT1.03 plasmid DNA were added to 10-cm dishes. The cells were incubated at 37 °C for 6 h. Then, the cells were further incubated with a culture medium for 18 h.

### Monitoring of cells transfected with ATeam

Transfected cells were passaged to 35-mm GLASS BASE DISH (IWAKI, 3961–035) (2 × 10^5^ cells/2 mL of culture medium). Immediately before observation, the medium was replaced with PBS at 37 °C. Cells were monitored by LSM780 (Carl Zeiss) (excitation: 405 nm, emission: 440–500 nm [CFP], 500–570 nm [YFP]). The YFP/CFP emission ratio was calculated by dividing pixel-by-pixel YFP images with CFP images. Pseudo-color was generated using ImageJ software (ImageJ bundled with 64-bit Java 1.8.0_172, https://imagej.nih.gov/ij/download.html).

### Statistical analysis

All data are presented as mean ± SE. Pair-wise comparisons were made using the Student’s t-test. Comparisons among multiple treatments were performed using a one-way analysis of variance (ANOVA), followed by an appropriate post hoc test. P values (both sides) were considered significant at P < 0.05. Statistical analyses were performed using GraphPad Prism 5.0 (San Diego, CA, USA).

## Results

**Figure 1:**
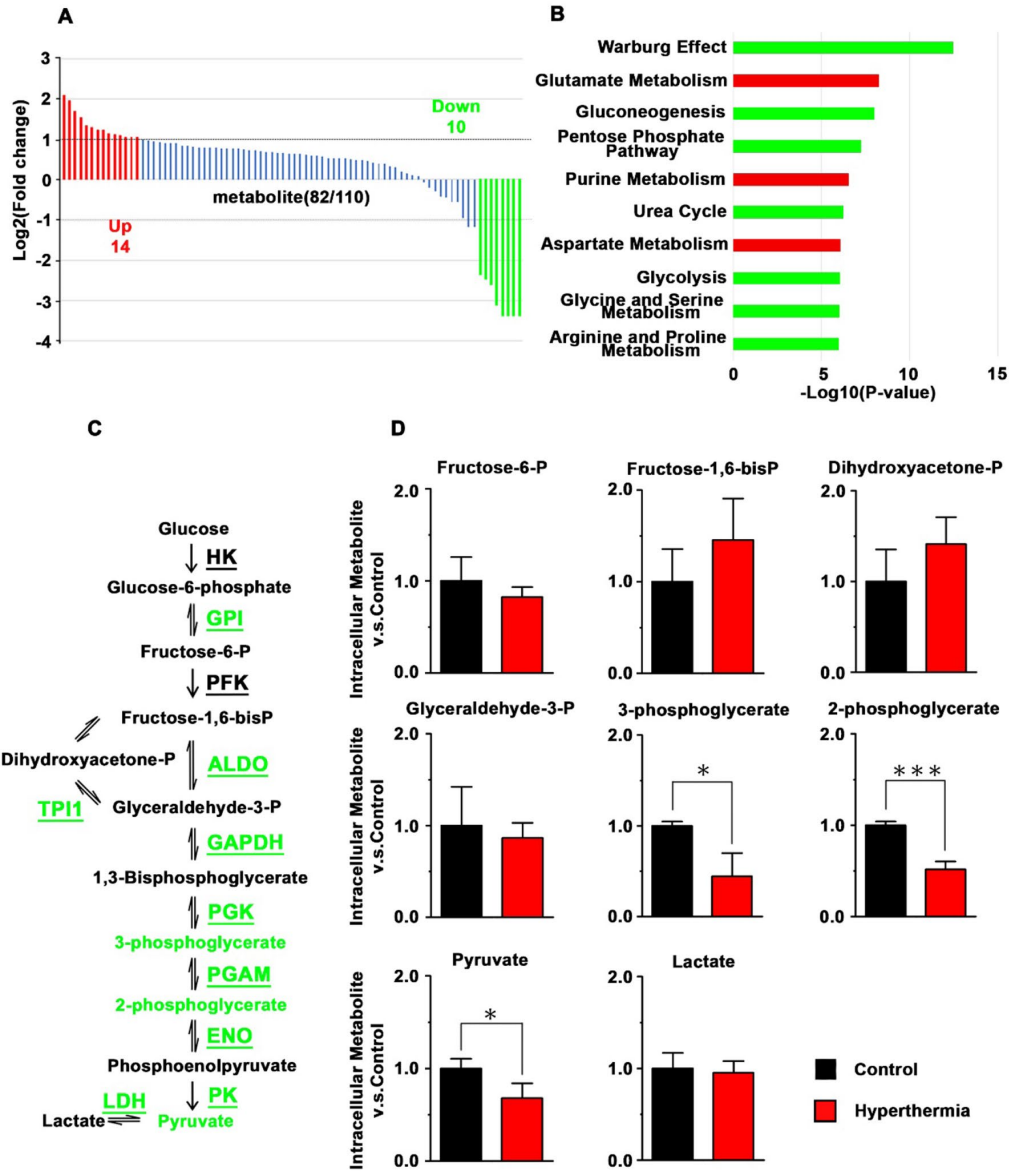
Analysis of Metabolites in heated SKOV3 cells. (**A**) Log2 fold change of metabolites in SKOV3 cells after hyperthermia, as compared to control SKOV3 cells. Significant change according to a fold change greater than 2 is indicated by colors (red: upregulated, green: downregulated). (**B**) Enriched metabolic pathways identified for the affected metabolites in SKOV3 after hyperthermia. Green and Red: Predicted as downregulated and upregulated pathways, respectively. (**C**) Schematic of the glycolysis pathway. Enzymes are underlined. Enzymes and metabolites highlighted in green represent downregulated proteins and metabolites in SKOV3 cells after hyperthermia. (**D**) Relative amounts of metabolites involved in glycolysis. Average ± S.D.; n = 3. *P < 0.05; ***P < 0.001.

Ovarian SKOV3 and uterine KLE cells showed resistance to hyperthermia compared to A2780 and Hec-1A (Fig. S1). The time profiles of SKOV3 cell viability were also clearly different from those of A2780 cells (Fig. S2). Thus, SKOV3 and KLE were identified as hyperthermia-resistant cells. We previously demonstrated that glycolysis enzymes were downregulated explicitly in hyperthermia-resistant SKOV3 cells after hyperthermia compared to hyperthermia-sensitive A2780 cells^8^. However, the fluctuation of these metabolites is yet to be investigated. Thus, we measured 110 metabolites in SKOV3 cells treated with CE-TOFMS after hyperthermia (Fig. 1A). Among the 82 metabolites that were successfully quantified using CE-TOFMS (Table S1), 14 and 10 metabolites were upregulated and downregulated, respectively, in heated SKOV3 cells compared to the control (Fig. 1A). These 24 metabolites were analyzed using MetaboAnalyst to identify the affected metabolic pathways. The top-ranked pathways included glucose metabolism-related pathways, such as the Warburg effect, glycolysis, and gluconeogenesis, which were predicted to be downregulated (Fig. 1B). Because a remarkable decrease in glycolytic enzymes was observed in SKOV3 cells after hyperthermia in a previous study^8^, we further examined metabolites in the glycolysis pathway (Fig. 1C). The change in metabolites was consistent with the downregulation of glycolytic enzymes after thermal stimulation. Although the upstream metabolites of the glycolysis pathway were unaffected, a significant decrease was observed in the levels of downstream metabolites, such as 2-phosphoglycerate, 3-phosphoglycerate, and pyruvate (Fig. 1D). The analysis of metabolites revealed that the changes in metabolites were consistent with the downregulation of glycolytic enzymes after hyperthermia in hyperthermia-resistant SKOV3 cells.

**Figure 2:**
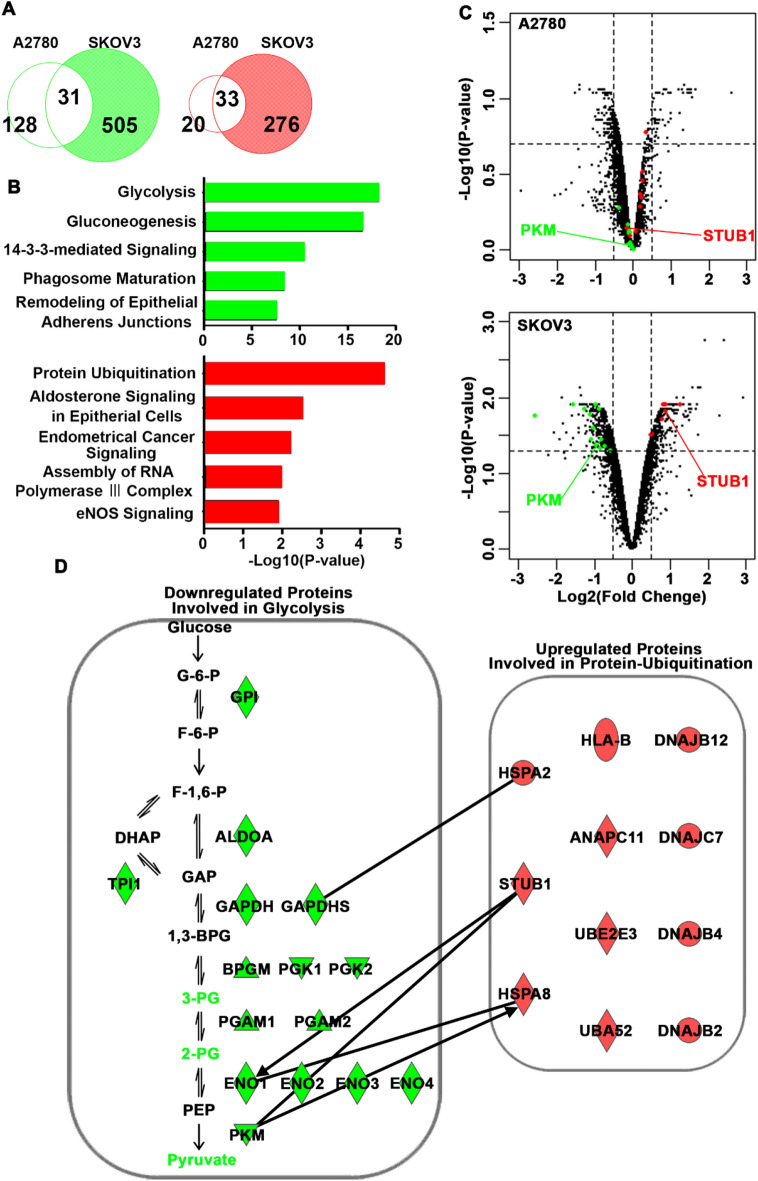
Detailed proteomics analysis of heated SKOV3 cells. (**A**) Venn diagrams of downregulated proteins (left) and upregulated proteins (right) in A2780 and SKOV3 cells after hyperthermia. Data were adapted from Hatakeyama H^8^. (**B**) Graphs show the top five enriched GO annotations for biological processes for differentially expressed downregulated proteins (upper) and upregulated proteins (lower) in SKOV3 cells. (**C**) Volcano plots of differentially expressed proteins in A2780 (upper) and SKO3 (lower) cells after hyperthermia. Proteins associated glycolysis and protein ubiquitination were represented as green and red dots. (**D**) Potential connections between glycolysis-related and ubiquitination-related proteins. Red and green indicate upregulation and downregulation, respectively. The lines indicate binding of two gene products, and lines terminating with arrows indicate one gene product acting on another gene product.

Next, we investigated the regulation of glycolytic enzymes after hyperthermia in SKOV3 cells. Proteomic data adapted from a previous report were further analyzed. After hyperthermia, downregulated proteins (53 proteins in A2780 cells and 309 proteins in SKOV3 cells, respectively) and upregulated proteins (159 proteins in A2780 cells and 536 proteins in SKOV3 cells) were extracted. To identify the specific alterations in proteins in SKOV3 cells, we excluded overlapping proteins between A2780 and SKOV3 cells because of the typical response to hyperthermia. Of the 536 downregulated proteins, 505 were selectively downregulated in SKOV3 cells, while 276 of 309 upregulated proteins were selectively upregulated in SKOV3 cells (Fig. 2A). In the pathway analysis with DAVID, glucose metabolism-related pathways such as glycolysis and gluconeogenesis were ranked the highest based on the downregulated proteins, a result that is consistent with the findings of a previous study^8^ (Fig. 2B). In contrast, the proteins involved in the ubiquitination pathway were found to be most upregulated in SKOV3 cells (Fig. 2B). We confirmed that ubiquitination-associated and glycolysis-associated proteins were not affected in A2780 cells after hyperthermia (Fig. 2C). Therefore, we hypothesized that the ubiquitination pathway degrades glycolytic enzymes after hyperthermia in SKOV3 cells. We explored the connection between downregulated proteins involved in glycolysis and upregulated proteins involved in ubiquitination using IPA. The analysis showed that the E3 ubiquitin ligase STUB1 binds to PKM (Fig. 2D).

**Figure 3:**
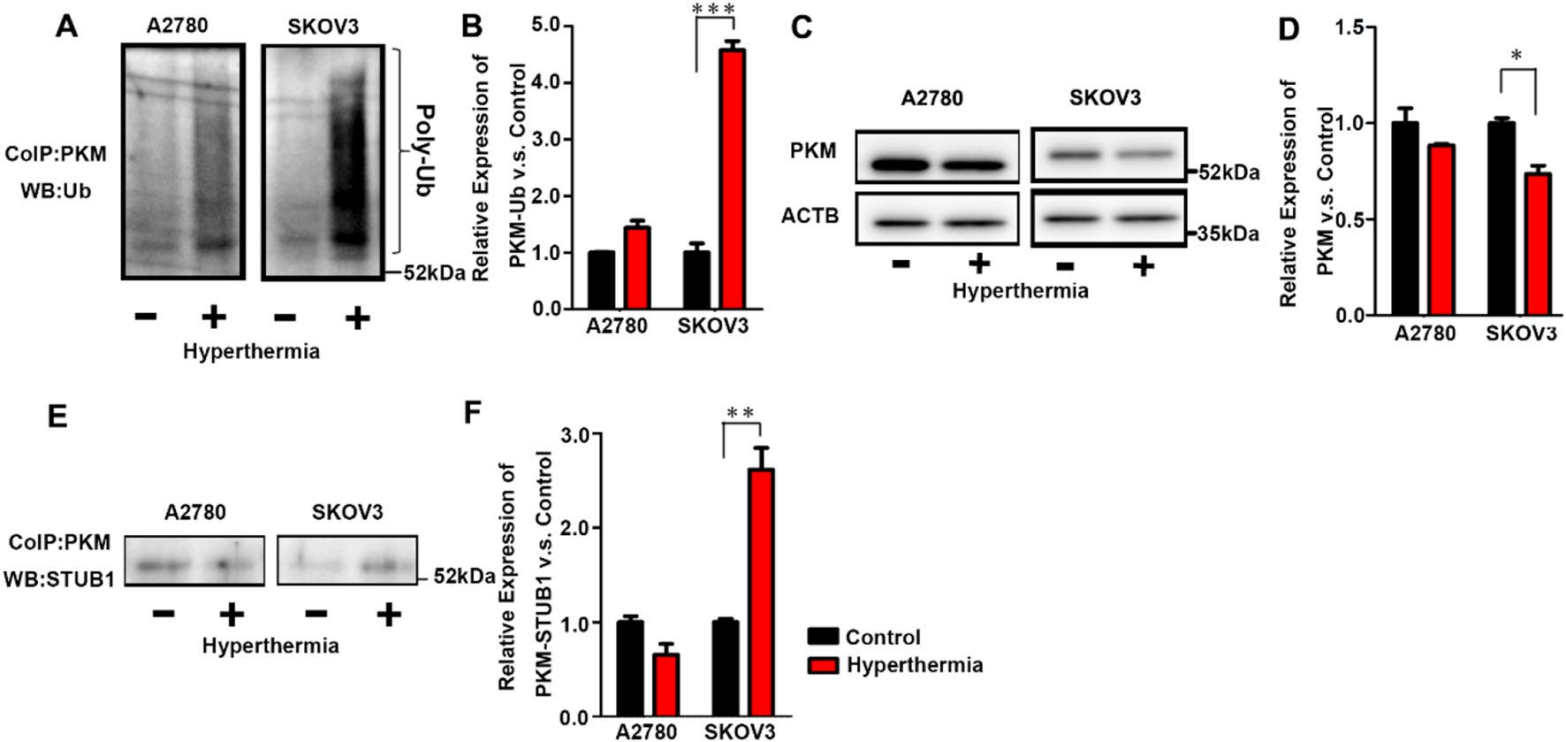
Ubiquitination and degradation of PKM. (**A**) Western blot analysis of ubiquitin after co-immunoprecipitation of PKM in A2780 and SKOV3 cells treated with hyperthermia (46 °C, 1 h). (**B**) Quantitative analysis of Ubiquitinated PKM1/2. (**C**) Western blot analysis of PKM1/2 in A2780 and SKOV3 cells treated with hyperthermia (46 °C, 1 h). (**D**) Quantitative analysis of PKM1/2. (**E**) Western blot analysis of STUB1 after co-immunoprecipitation with PKM1/2 in A2780 and SKOV3 cells treated with hyperthermia (46 °C, 1 h). (**F**) Quantitative analysis of PKM1/2 binding STUB1. Average ± S.D.; n = 3. *P < 0.05; **P < 0.01; ***P < 0.001. N.S.: Not significant difference.

Next, we examined the ubiquitination of PKM1/2 after hyperthermia through co-immunoprecipitation and western blotting. Since the gene expression of *HSP70* increased over 4 h after hyperthermia (Fig. S3), the response to hyperthermia occurred over 4 h after hyperthermia in SKOV3 cells. Thus, the samples were harvested 4 h after hyperthermia to elucidate the mechanisms. After hyperthermia, the amount of ubiquitinated PKM1/2 was found to be significantly increased in SKOV3 cells (Fig. 3A,B). We also measured the total PKM1/2 after hyperthermia using western blotting (Fig. 3C,D). The amount of total PKM1/2 was found to be decreased after hyperthermia in SKOV3; no change was observed in A2780, corresponding to the proteomics data^8^. The amount of STUB1 bound to PKM was also significantly increased in SKOV3 cells (Fig. 3E,F). These results demonstrated that the amount of PKM1/2, a glycolytic rate-limiting enzyme, was decreased by ubiquitination in hyperthermia-resistant SKOV3 cells after hyperthermia.

**Figure 4:**
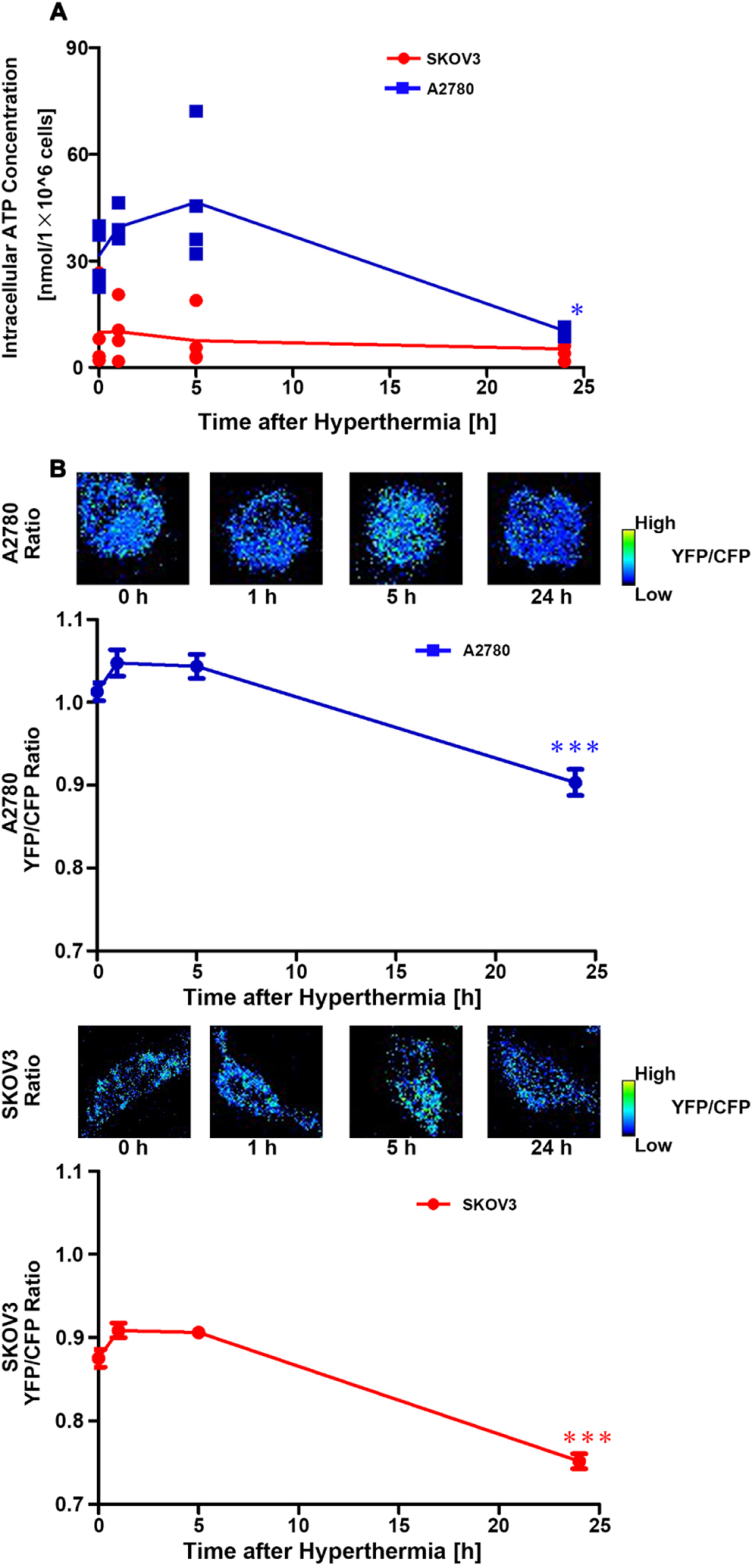
Quantification of intracellular ATP in A2780 and SKOV3 cells treated with hyperthermia. (**A**) Time profiles of ATP concentrations in A2780 and SKOV3 cells. Cells were treated with hyperthermia at 46 °C for 1 h (between 0 and 1 h) and then incubated at 37 °C until indicated time points. Intracellular ATP was quantified with CE-TOFMS. Means and observation points were plotted. Monitoring of cytoplasmic ATP levels of A2780 (upper) and SKOV3 (lower) cells using AT1.03. Cells that were transfected with AT1.03 were treated with hyperthermia at 46 °C for 1 h (between 0 and 1 h) and then incubated at 37 °C until indicated time points. Sequential wide-field images of YFP/CFP emission ratio and time courses of averaged YFP/CFP emission ratio of A2780 (upper) and SKOV3 (lower) cells expressing AT1.03. Mean ± S.E. (n = 40). *P < 0.05; ***P < 0.001 (vs 0 h).

Generally, cancer cells produce ATP through glycolysis-lactate fermentation, which is recognized as the Warburg effect, to gain energy^13^. Therefore, it was predicted that the amount of ATP in SKOV3 cells would decrease after hyperthermia treatment. Thus, we measured the changes in ATP concentrations in A2780 and SKOV3 cells over a 24-h period after hyperthermia, using CE-TOFMS and AT1.03 transfection (Fig. 4). The amount of ATP in A2780 cells was found to be higher than that in SKOV3 cells at the base and tended to increase after hyperthermia before decreasing over the 24-h period in both cell types (Fig. 4A). Contrary to our expectation, no significant decrease in ATP levels was observed in SKOV3 cells after hyperthermia. These trends were also confirmed using AT1.03, which binds to ATP and triggers fluorescence resonance energy transfer (FRET) between CFP and YFP, increasing the ratio of YFP to CFP. As shown in Fig. 4B, the ratio of YFP to CFP was comparable until 5 h and then decreased at 24 h.

**Figure 5:**
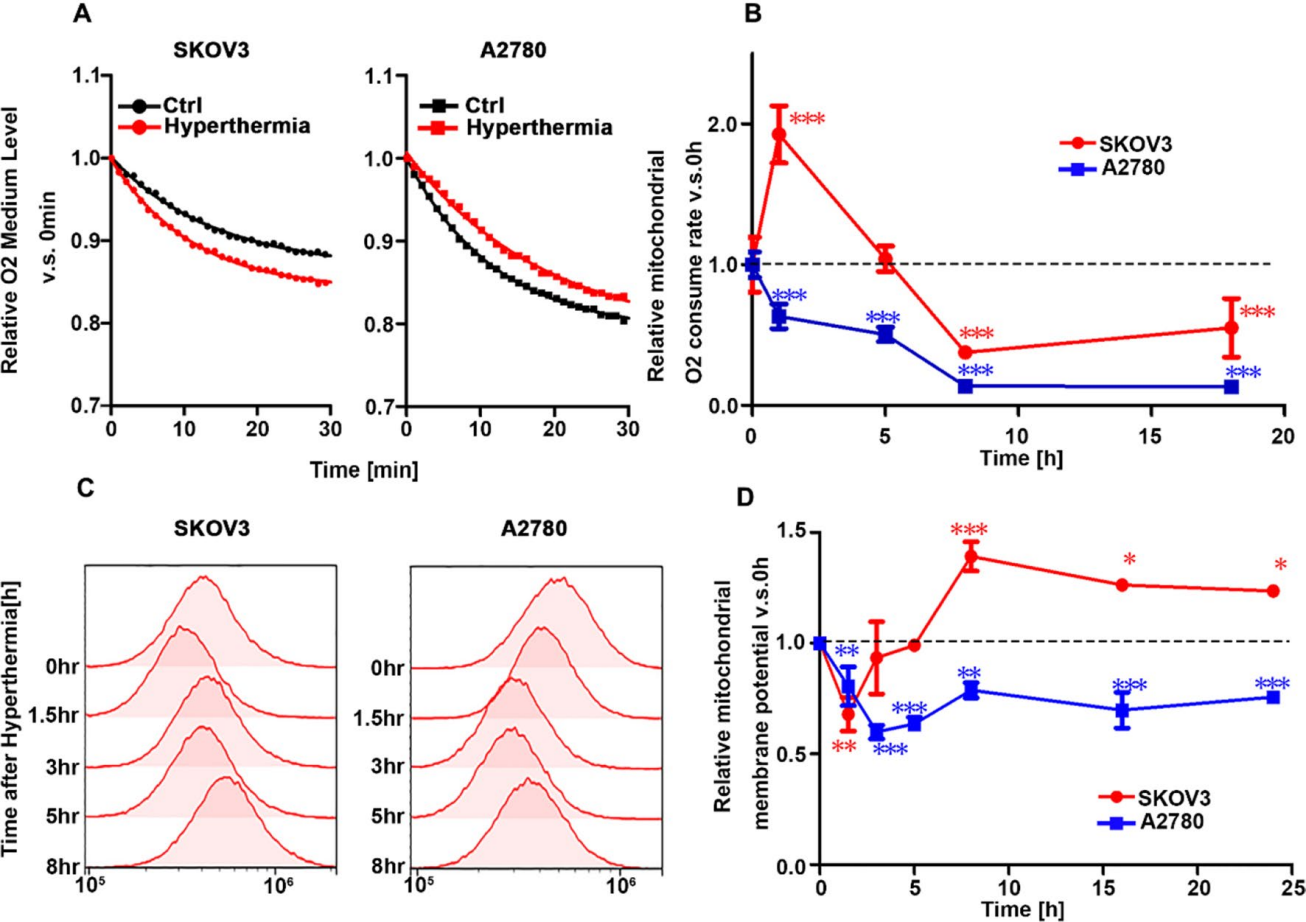
O_2_ consumption and mitochondrial membrane activity of A2780 and SKOV3 cells treated with hyperthermia. (**A**) Relative amount of O_2_ in the medium vs. time 0. A2780 and SKOV3 cells were treated with hyperthermia. (**B**) Time profiles of O_2_ consumption rates in A2780 and SKOV3 cells treated with hyperthermia. (**C**) Mitochondrial membrane activities in heated A2780 and SKOV3 cells stained with Rhodamine 123 were measured using flow cytometry. (**D**) Time profiles of membrane potentials of mitochondria in A2780 and SKOV3 cells treated with hyperthermia (0 to 1 h). Mean ± S.D. (n = 3). *P < 0.05;**P < 0.01; ***P < 0.001 (vs 0 h).

Although the glycolysis pathway was suppressed in SKOV3 cells, a decrease in ATP levels was not observed. This suggests that ATP is produced through an alternative pathway instead of glycolytic-lactate fermentation in SKOV3 cells after hyperthermia. We hypothesized that mitochondria produce ATP under heat stress conditions. Thus, we measured indicators of mitochondrial activity, such as O_2_ consumption and membrane potential, under hyperthermia in A2780 and SKOV3 cells (Fig. 5). The O_2_ consumption decreased after hyperthermia in A2780 cells but increased in SKOV3 cells (Fig. 5A). The amount of O_2_ in the medium was measured using a sensor dish reader (Fig. 5A). There was no difference in the concentration of O_2_ in media without cells before and after hyperthermia (Fig. S4), which confirmed that the change in oxygen consumption after hyperthermia was caused by the response of these cells to hyperthermia. The O_2_ consumption rate was calculated using the slope at time 0. The O_2_ consumption rate in A2780 dropped immediately after hyperthermia and could not recover until 24 h.

In contrast, the O_2_ consumption rate in SKOV3 cells significantly increased after hyperthermia treatment (Fig. 5B). Mitochondrial membrane potential was measured using Rhodamine 123 and measured using a flow cytometer (Fig. 5C). The mitochondrial membrane potential in A2780 decreased after hyperthermia and did not recover to the level before hyperthermia until 24 h. However, the mitochondrial membrane potential in SKOV3 cells decreased after hyperthermia despite the increased O_2_ consumption, which indicated that the proton gradient generated by the respiratory chain was consumed via ATPase (Fig. 5B). Because 2-Deoxy-D-glucose (2-DG), a competitive inhibitor of glucose, induces oxidative phosphorylation instead of aerobic glycolysis^14^, we assessed the effect of the metabolic shift induced by 2-DG. No tolerance to hyperthermia was observed at 2780 (Fig. S5), indicating that the induction of thermoresistance requires metabolic adaptation from glycolysis to phosphorylation, accompanied by upregulation of mitochondrial activity. Mitochondrial activation was also observed in hyperthermia-resistant uterine KLE cells after hyperthermia but not in sensitive Hec-1A cells (Fig. S6). These results indicated that mitochondria in SKOV3 cells were activated after hyperthermia, which might have compensated for the loss of ATP production due to the suppression of glycolysis-lactate fermentation after hyperthermia.

**Figure 6:**
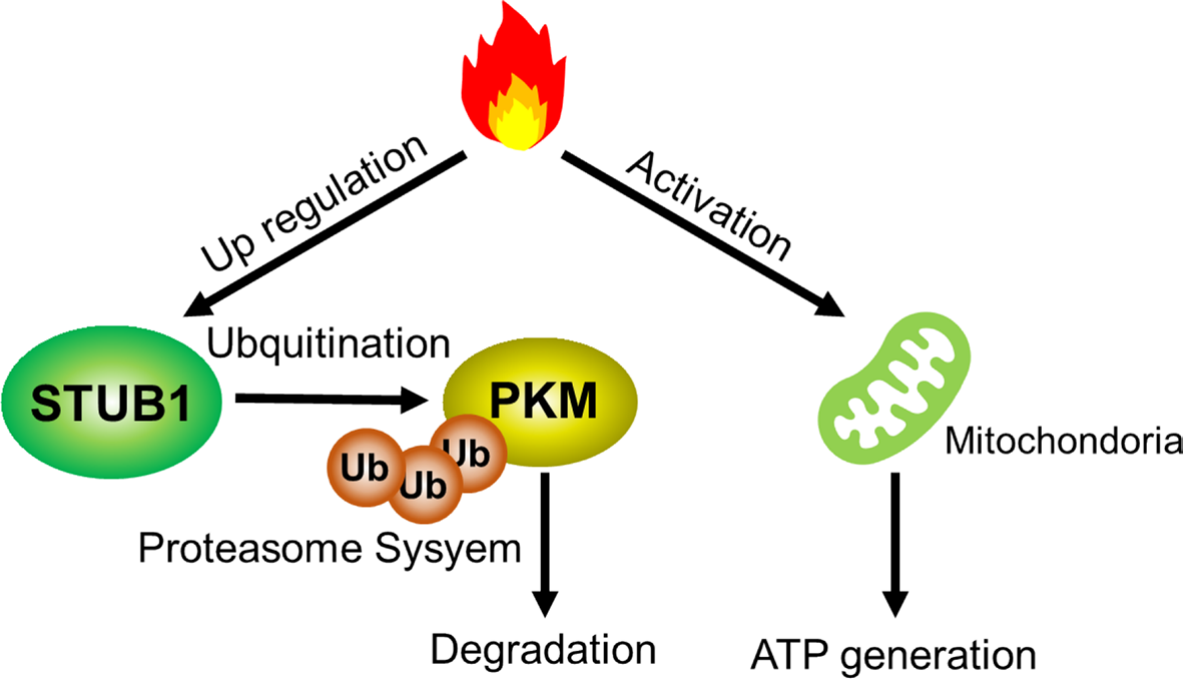
A proposed mechanism of ATP metabolic adaptation in response to hyperthermia in resistant SKOV3 cells. Hyperthermia upregulates the STUB1 E3 ligase in hyperthermia resistant ovarian SKOV3 cells. STUB1 causes degradation of a glycolysis enzyme, PKM1/2, through the ubiquitin proteasome system, causing inhibition of glycolytic activity. On the other hand, mitochondrial activity is upregulated in response to hyperthermia, which promotes ATP production.

In conclusion, when exposed to hyperthermia, SKOV3 cells show metabolic adaptation to heat stress. Our results suggest that A2780 cells do not suppress glycolysis because of the decreased activity of mitochondria. In contrast, SKOV3 cells suppressed glycolysis-lactate fermentation through ubiquitin-mediated degradation of PKM1/2 and activated mitochondria (Fig. 6). The metabolic shift in mitochondria reimburses the decrease in ATP production due to the suppression of glycolysis-lactate fermentation in ovarian SKOV3 cells.

## Discussion

We reported that the sensitivity of ovarian and uterine cancer cells to hyperthermia varies across cell lines^8^. Through proteomic analysis, we found that enzymes involved in glycolysis were degraded after hyperthermia in hyperthermia-resistant SKOV3 cells. However, the detailed mechanisms by which these enzymes are degraded after hyperthermia in resistant SKOV3 cells have not yet been investigated. Further analysis of the proteomic data revealed that the proteins involved in the ubiquitination pathway were explicitly upregulated in SKOV3 cells after hyperthermia. Therefore, we hypothesized that some enzymes involved in glycolysis are degraded via ubiquitination. Pathway analysis using IPA predicted that an E3 ligase, STUB1, could ubiquitinate PKM. As expected, the level of PKM1/2 ubiquitination was enhanced, and a decrease in PKM1/2 was observed in SKOV3 cells after hyperthermia. Our findings are supported by a previous study, which found that the levels of PKM2 decreased after the overexpression of STUB1 in cancer cells, which resulted in suppression of the Warburg effect^15^. It has been reported that STUB1 degrades target proteins when STUB1 associates with HSP70^16^, and there is a possibility that upregulated HSP70 in response to hyperthermia stabilizes and/or activates STUB1. Although the precise mechanism behind the increase in STUB1 expression in response to hyperthermia remains to be investigated, PKM1/2 was degraded presumably through ubiquitination by STUB1. These results suggest that STUB1 is an upstream regulator of metabolic adaptation in response to hyperthermia. It has been reported that other enzymes involved in glycolysis are also degraded through ubiquitination in cancer cells^17,18^. It is possible that the degradation of these other enzymes also depends on ubiquitination during heat stress.

The levels of ATP in A2780 cells were maintained until 5 h, presumably because the expression of enzymes involved in glycolysis was not affected by hyperthermia, and ATP production through the Warburg effect might have been unaffected as well. However, the ATP levels at 24 h after hyperthermia dropped below the levels before hyperthermia, which is consistent with the findings of a previous study, in which a decrease in ATP levels was observed in hepatocellular carcinoma cells at 24 h after hyperthermia^19^. Thus, we expected that the intracellular ATP level would decrease in SKOV3 cells because of the suppression of the glycolysis pathway. However, the levels of intracellular ATP did not decrease in SKOV3 cells 5 h after hyperthermia. We hypothesized that the decrease in ATP production due to the suppression of glycolysis is compensated for by another metabolic pathway. Therefore, we compared the mitochondrial activities of A2780 and SKOV3 cells after hyperthermia treatment. The results demonstrated that mitochondrial activity in SKOV3 cells significantly increased during hyperthermia. The major pathway of ATP production was likely shifted from the Warburg effect to oxidative phosphorylation in mitochondria in SKOV3 cells in response to hyperthermia. It has been reported that the metabolic shift from the Warburg effect to oxidative phosphorylation in cancer cells occurs under glucose exhaustion and acquired drug resistance^12,20,21^. To the best of our knowledge, this is the first report to show that hyperthermia induces energy metabolic adaptation from the Warburg effect to oxidative phosphorylation. The metabolites in the TCA cycle should be compensated for by pathways other than glycolysis. We hypothesized that glutaminolysis might contribute to providing α-keto glutamate to the TCA cycle instead of acetyl-CoA from glycolysis^20^. Analysis of the flux of metabolites would reveal how production and consumption of ATP are maintained under glycolysis-suppressed conditions^22,23^.

The physiological significance of metabolic adaptation in response to hyperthermia has not yet been elucidated. Heat production is a by-product of glycolytic metabolic activity of glycolysis^24^. In contrast, mitochondria in brown adipocytes are known to be involved in thermogenesis^25,26^. In brown adipocytes, uncoupling protein 1 (UCP1) uncouples the proton gradient of the mitochondrial membrane with the activity of ATP synthase, resulting in heat generation instead of ATP production^27^. Because no expression of UCP1 protein was observed in SKOV3 cells^8^, it is likely that oxidative phosphorylation in mitochondria in response to hyperthermia mainly produces ATP along with a decrease in mitochondrial membrane potential, without thermal generation through UCP uncoupling. Taken together, these findings indicate that the energy metabolic adaptation in response to hyperthermia results in less heat generation in SKOV3 cells, which leads to high tolerability to hyperthermia. Real-time intracellular temperature measurements^28^ should consider this issue.

Regional hyperthermia, such as HIPEC, is currently used for cancer treatment. Significant progress has been made in developing strategies to generate heat via exogenous stimuli-responsive nanocarriers to light-induced activation, magnetic field, and ultra sound^29^. In a previous study, knocking down key molecules sensitized ovarian cancers to local hyperthermia^8,30^. This study proposes potential targets for the sensitization to hyperthermia. The ubiquitin inhibitor PYR-41 sensitized SKOV3 cells to hyperthermia (data not shown). There is a possibility that the specific knockdown of STUB in hyperthermia-resistant tumors sensitizes them to hyperthermia. We also expect that the inhibition of metabolic adaptation will have a more dominant effect on the sensitization of cancer cells to hyperthermia because metabolomics is closely linked to the functional phenotype^31^. Combining this strategy with the inhibition of the targets would provide effective hyperthermia treatment and improve clinical outcomes for ovarian and uterine cancer patients.

## Supplementary Information

Supplementary Information 1.

Supplementary Information 2.

## Data Availability

The data for this manuscript can be obtained from the author upon reasonable request.
