# Supplementary material for: Investigation of energy metabolic dynamism in hyperthermia-resistant ovarian and uterine cancer cells under heat stress
Source: Sci Rep. 2021 Jul 19;11:14726. doi: 10.1038/s41598-021-94031-9 (PMC8289900; doi:10.1038/s41598-021-94031-9)
Supplement: Supplementary file 2 — Supplementary Information 2. [file 41598_2021_94031_MOESM2_ESM.pdf]

Supplemental Information

Figure S1 Hyperthermia sensitivity of ovarian and uterine cells
Figure S2 Time profiles of the viability of hyperthermia sensitive and resistant cells
Figure S3 The expression of *HSP70* in SKOV3 after hyperthermia
Figure S4 The impact of increased temperature on oxygen content in the medium
Figure S5 The effect of inhibition of glycolysis on hyperthermia resistance
Figure S6 Mitochondrial membrane activity of Hec-1A and KLE cells treated with hyperthermia
Figure S7 Western blotting original membrane images

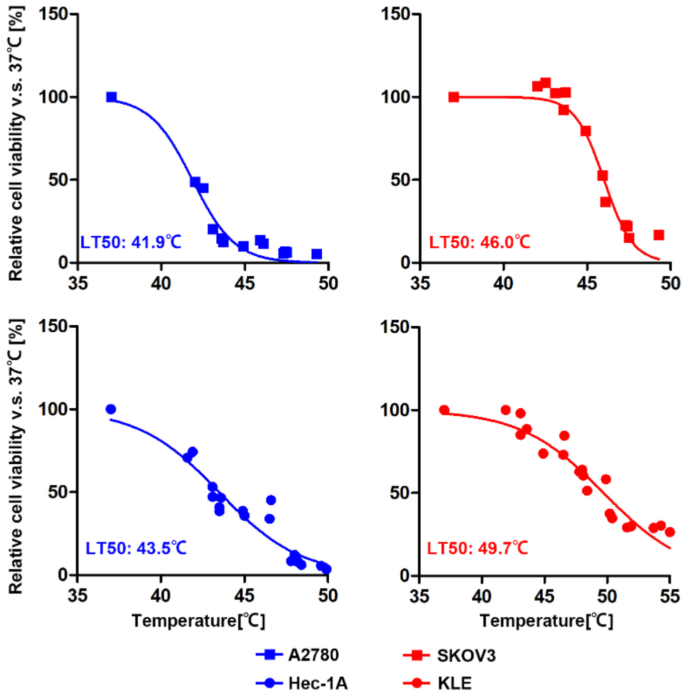

Figure S1. Hyperthermia sensitivity of ovarian and uterine cells.

Ovarian A2780 and SKOV3 cells and uterine Hec-1A and KLE cells were treated with hyperthermia at various temperatures for 1 h and then incubated at 37 °C. Cell viability was determined using an MTT assay after 24 h. The value in each graph represents LT50, which produced 50% cell death. The LT50s of SKOV3 and KLE cells were higher than those of A2780 and Hec-1A cells. The results confirmed that SKOV3 and KLE cells were hyperthermia-resistant. The results were consistent with those of our previous report (Hatakeyama H, et al. Cell Rep, 17, 1621-1631, 2016).

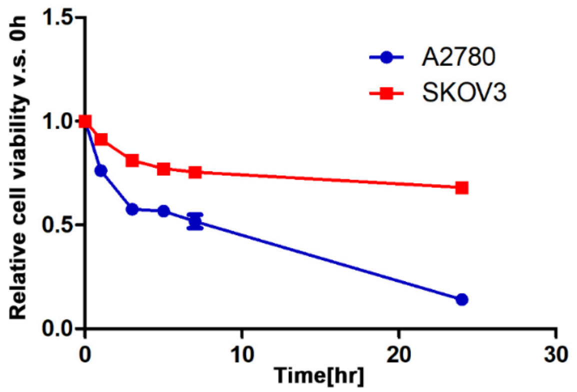

Figure S2. Time profiles of the viability of hyperthermia sensitive and resistant cells. A2780 and SKOV3 cells were treated with hyperthermia at 46 °C for 1 hr and then incubated at 37 °C. Cell viability was determined using an MTT assay at indicated time points. The time-profile of cell viability of A2780 was clearly different from that of SKOV3, which demonstrated that hyperthermia sensitivity varied among A2780 and SKOV3 cells.

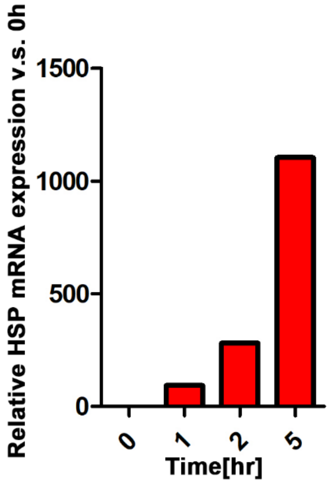

Figure S3 The expression of *HSP70* in SKOV3 after hyperthermia.

SKOV3 cells were treated with hyperthermia at 46°C for 1 h, followed by incubation at 37°C for up to 5 h. Cells were harvested, and *HSP70* (*HSPA6*) expression was determined by qRT-PCR. RNA was isolated from cultured cells using RNAzol (Molecular Research Center, Cincinnati, OH, U.S.A.) according to the manufacturer's protocol. RNA quality and quantity were assessed using a NanoDrop One (Thermo Fisher Scientific, Wilmington, DE, U.S.A.). cDNA was synthesized from 1000 ng of RNA by using a ReverTra Ace qPCR RT Master Mix (TOYOBO, Osaka, Japan) following the manufacturer's instructions. Analysis of mRNA expression in the cells was performed using a StepOne Real Time PCR System (Applied Biosystems, Foster City, CA, U.S.A.) with THUNDERBIRD SYBR qPCR Mix (TOYOBO). Each PCR cycle consisted of 5 s of denaturation at 95°C and 31 s of annealing and extension at 60°C (40 cycles). The amount of target gene in the cells was calculated using the ΔΔCt method, normalized according to β-actin (*ACTB*) mRNA. Primers used in qRT-PCR were as follows:

HSPA6

Forward 5´-CGCTGCGAGTCATTGAAATA-3´

Reverse 5´-GAGATCTCGTCCATGGTGCT -3´

ACTB

Forward 5´-TTCAACACCCCAGCCATGTACG-3´

Reverse 5´-GTGGTGGTGAAGCTGTAGCC -3´

The gene expression at 0 h represents *HSP70* expression in SKOV3 cells before hyperthermia.

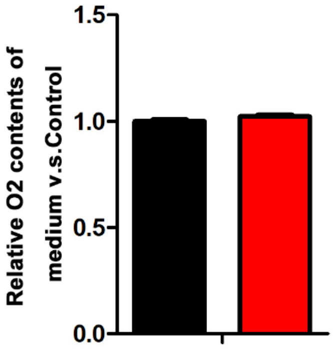

Figure S4 The impact of increased temperature on oxygen content in the medium. Oxygen amount in the medium without cells was measured before and after hyperthermia at 46 °C for 1 hr. Black and red represent before and after hyperthermia. There was no impact of hyperthermia on oxygen content in the medium. Average ± S.D.; n = 3.

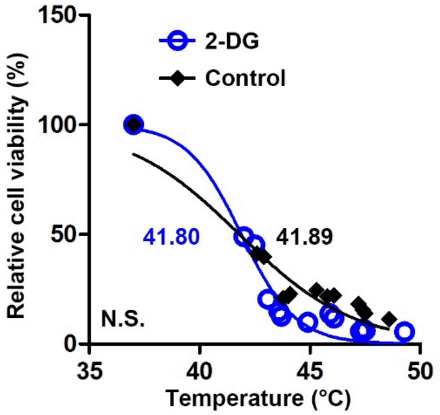

Figure S5. The effect of inhibition of glycolysis on hyperthermia resistance. To examine the effect of induction of oxidative phosphorylation instead of aerobic glycolysis by treatment with 2-Deoxy-D-glucose (2-DG), a competitive inhibitor of glucose (Sottonik J. Clinical & Experimental Metastasis, 28(8),865-875, 2011). A2780 cells were treated with 5 mM 2-DG and hyperthermia at various temperatures for 1 hour and further incubated at 37°C, then evaluated cell viability at 24 hr. LT50 was not affected by treatment with 2-DG. These results suggested that the induction of thermoresistance requires metabolic adaptation from glycolysis to phosphorylation accompanied by up-regulation of mitochondrial activity. N.S.: Not significant difference in LT50 between control and 2-DG.

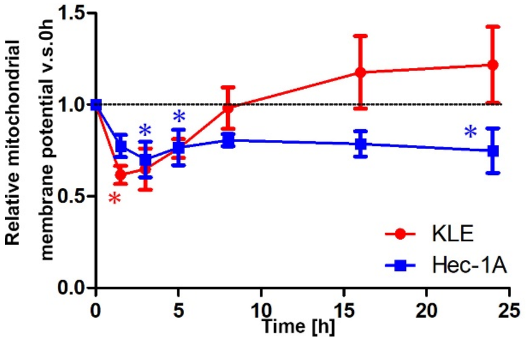

Figure S6 Mitochondrial membrane activity of Hec-1A and KLE cells treated with hyperthermia. Mitochondrial membrane activities in heated Hec-1A and KLE cells stained with Rhodamine 123 were measured using flow cytometry. Time profiles of membrane potentials of mitochondria in Hec-1A and KLE cells treated with hyperthermia (0 to 1 h). Mean ± S.D. (n = 3).

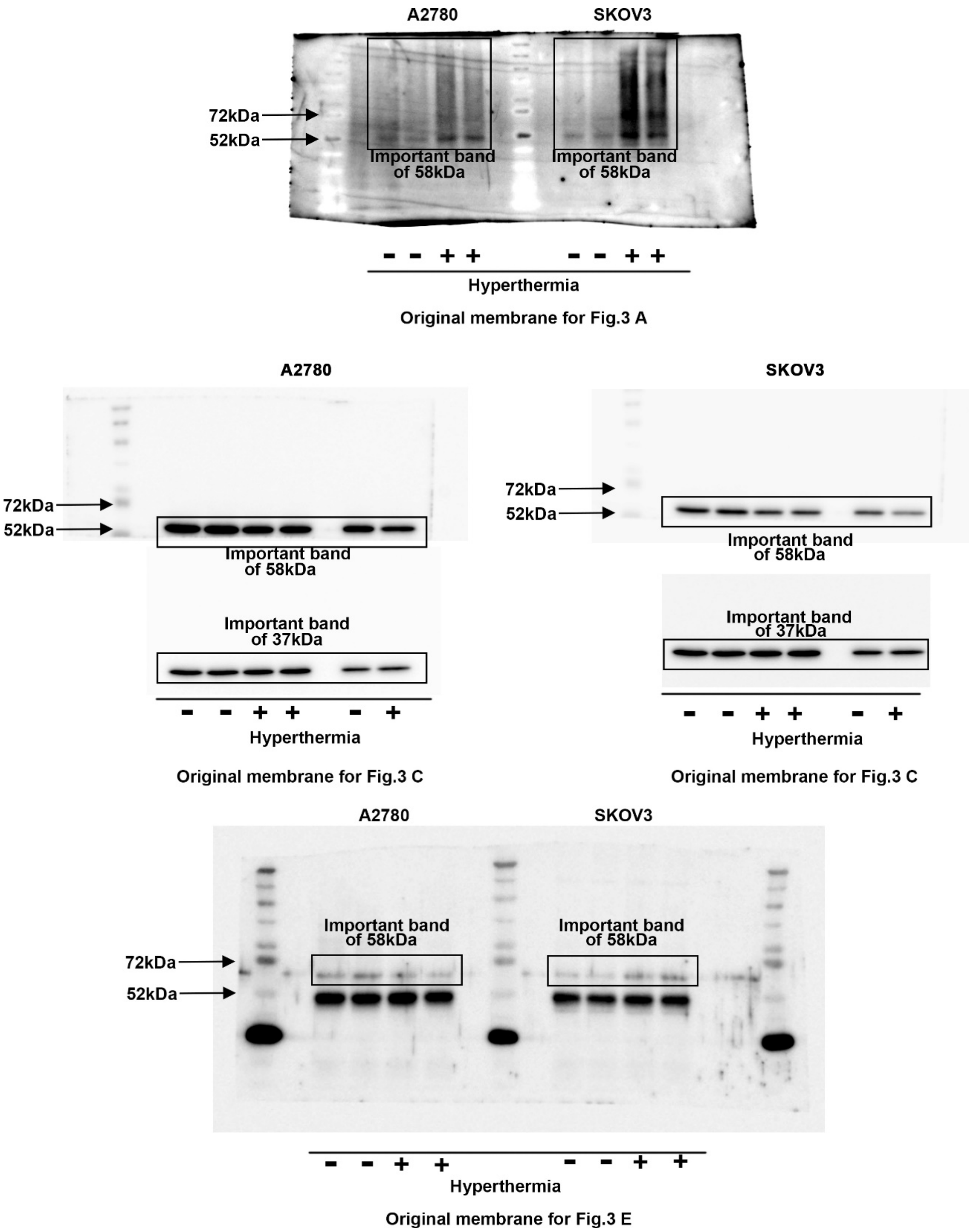

Figure S7. Western blotting original membrane images.

(A) Original membrane for Figure 3A. (B) Original membranes for Figure 3C. (C) Original membrane for Figure 3E. Unprocessed membrane images are represented in the next page.

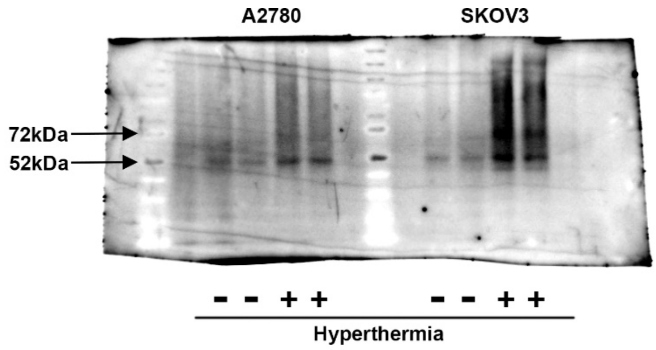

Original membrane for Fig.3 A

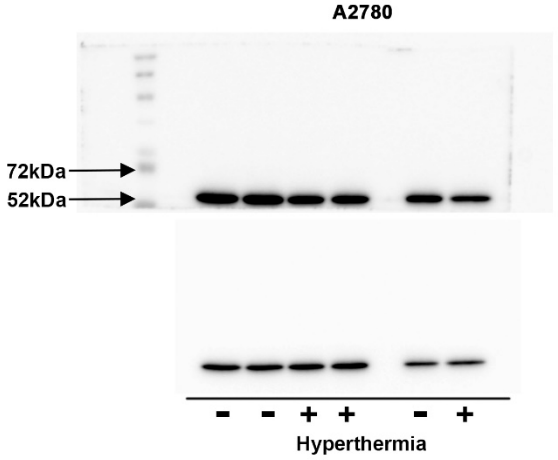

Original membrane for Fig.3 C

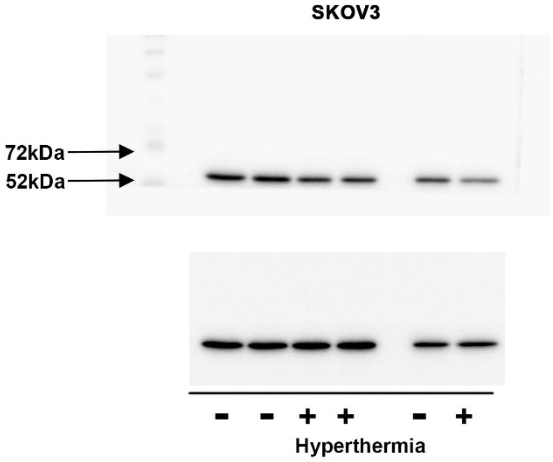

Original membrane for Fig.3 C

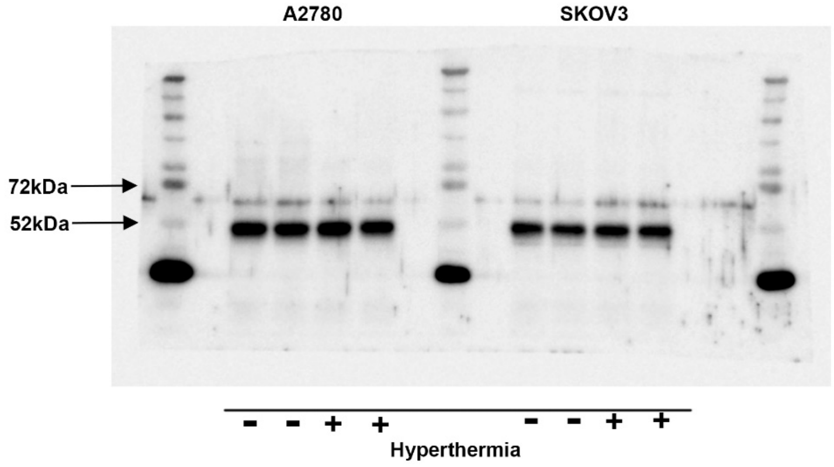

Original membrane for Fig.3 E
